# Association between progression of knee osteoarthritis pathology and gait changes over two years: Data from the IMI-APPROACH cohort

**DOI:** 10.1016/j.ostima.2024.100232

**Published:** 2024-06-10

**Authors:** Mylène P. Jansen, Diana Hodgins, Simon C. Mastbergen, Margreet Kloppenburg, Francisco J. Blanco, Ida K. Haugen, Francis Berenbaum, Felix Eckstein, Frank W. Roemer, Wolfgang Wirth

**Affiliations:** a Department of Rheumatology & Clinical Immunology, University Medical Center Utrecht, Utrecht, the Netherlands; b Dynamic Metrics Limited, Codicote, United Kingdom; c Department of Rheumatology, Leiden University Medical Center, Leiden, the Netherlands; d Clinical Epidemiology, Leiden University Medical Center, Leiden, the Netherlands; e Grupo de Investigación de Reumatología (GIR), INIBIC – Complejo Hospitalario Universitario de A Coruña, SERGAS. Centro de Investigación CICA, Departamento de Fisioterapia y Medicina, Universidad de A Coruña, A Coruña, Spain; f Center for treatment of Rheumatic and Musculoskeletal Diseases (REMEDY), Diakonhjemmet Hospital, Oslo, Norway; g Department of Rheumatology, AP-HP Saint-Antoine Hospital, Paris, France; h INSERM, Sorbonne University, Paris, France; i Institute of Imaging and Functional Musculoskeletal Research, Center of Anatomy and Cell Biology & Ludwig Boltzmann Institute for Arthritis and Rehabilitation (LBIAR), Paracelsus Medical University, Salzburg, Austria; j Chondrometrics GmbH, Freilassing, Germany; k Quantitative Imaging Center, Department of Radiology, Boston University School of Medicine, Boston, MA, USA; l Department of Radiology, Universitätsklinikum Erlangen and Friedrich-Alexander-University Erlangen-Nürnberg (FAU), Erlangen, Germany

**Keywords:** Knee osteoarthritis, Gait, Progression, Imaging, Bone marrow lesion, Meniscal extrusion

## Abstract

**Objective:**

Gait alterations in knee osteoarthritis (OA) patients are potentially related to structural progression of joint tissues, of which some are modifiable. The current objective was to determine whether progression in individual OA pathologies is related to gait kinematic parameters in knee OA patients, and whether these changes are influenced by pain.

**Design:**

Range of motion (ROM) during gait, joint tissue pathologies and morphology from index knee radiographs and MRI, and WOMAC pain were collected at baseline and at two-years in the IMI-APPROACH clinical knee OA cohort. Principal component (PC) analysis was performed on two-year change in gait parameters; the resulting (first) PC was compared between progressors and non-progressors for each structural parameter. When the PC indicated differences between groups (*p* < 0.1), individual gait parameters were compared for that structural outcome. Statistically significant differences in individual gait parameters were corrected for pain change.

**Results:**

191 patients (age 66.5 ± 6.7; BMI 27.5 ± 4.8; 76 % female; 51 % Kellgren-Lawrence grade ≥2) were analyzed. The gait change PC differed between progressors and non-progressors for meniscal extrusion, bone marrow lesions (BMLs), and patellofemoral cartilage lesions. Further, meniscal extrusion progressors showed significantly more knee ROM and calf ROM decrease, BML progressors worsened more in thigh ROM, and patellofemoral cartilage lesion progressors improved in knee ROM. BML results were no longer significant after pain change adjustment (*p* = 0.054).

**Conclusions:**

Meniscal extrusion and BML progression are associated with gait worsening, though for BMLs the effect might be influenced by pain.

## Introduction

While it is known that knee osteoarthritis (OA) leads to worsening of gait kinematics, it is not fully understood whether these changes are solely due to pain increase or due to structural progression as well [1,2]. Certain baseline OA pathologies, specifically osteophytes and meniscal extrusion, previously showed associations with lower leg gait kinematics in people with clinical knee OA [3]. Similarly, it could be expected that worsening of these, or other OA pathologies could be associated with gait worsening over time. Identifying these pathologies would provide specific structural treatment targets that have the potential to influence patients’ symptoms, such as functional decline. Treating these specific pathologies could improve knee function, as reflected by gait, or at least prevent (further) deterioration.

As such, the purpose of this study was to determine whether progression in individual structural OA pathologies over two years is related to gait kinematic parameters in patients suffering from knee OA, and whether these associations are influenced by pain changes.

## Methods

### Participants

The IMI-APPROACH cohort included 297 participants with clinical knee OA (American College of Rheumatology (ACR) criteria) [4]. The inclusion process has been described previously [5]. In short, exclusion criteria included recent or scheduled knee surgery, current knee or hip prosthesis, secondary knee OA (due to e.g. severe leg deformity or inflammatory joint disease), contraindications for undergoing MRI, and generalized pain syndrome (e.g. fibromyalgia).

For each participant, their index knee was determined at screening, based on ACR criteria and pain severity. Participants were followed for two years, during which data including imaging of the index knee, gait measurements, and pain (Western Ontario and McMaster Universities Osteoarthritis Index (WOMAC), taken from the Knee Injury and Osteoarthritis Outcome Score (KOOS) questionnaire) were collected. [6]

### Gait evaluation

Gait was evaluated using the GaitSmart system. The system uses six inertial measurement units (IMU), each comprising three tri-axial accelerometers and three tri-axial gyroscopes, allowing for movement analysis in the sagittal and frontal plane. The sensors were attached to the participant's pelvis, thighs and calves and, after a 10 s stationary period for calibration, participants were asked to walk 15–20 m at their own speed and return. The IMUs were then removed and connected to the laptop for further analysis. The GaitSmart system has previously been validated in comparison with 3D gait analysis in an optical gait lab, showing reproducible results and no differences in knee range of motion (ROM) between the two methods [7,8].

For the current study, only the most relevant gait parameters, based on previous analyses in the IMI-APPROACH cohort, were selected for evaluation [3,9]. These were the range of motion (ROM) of the knee in stance phase, ROM of the knee in swing phase, ROM of the thigh, and ROM of the calf, all in degrees for the index leg at baseline and two years.

### Structural evaluation

For the evaluation of structural knee OA pathology, radiographs and MRI scans of the index knee were obtained at baseline and at two-year follow-up. Weight-bearing semi-flexed posterior-anterior radiographs were acquired according to the Buckland-Wright protocol [10]. The most affected tibiofemoral compartment (MAC; medial or lateral) was determined for all participants by two readers in consensus, based on characteristics used in OA scoring systems (joint space width (JSW), osteophytes, sclerosis), using radiographs at the latest follow-up moment (two years). Femorotibial (FT) structural pathology was evaluated in the MAC, since this was expected to impact gait the most. Further, baseline and two-year follow-up radiographs were scored using Kellgren-Lawrence (KL) grading, and analyzed using KIDA by one experienced observer [11,12]. The minimum JSW (mm), FT angle (degrees valgus), mean subchondral bone density (mm Aluminum equivalent (mm Al eq) in reference to an aluminum step wedge), and total osteophyte area (mm^2^) were evaluated for the MAC.

1.5T or 3T MRI scans were acquired, including sagittal 3D SPGR sequences for the quantitative analysis (qMRI) of cartilage thickness, and sagittal and coronal intermediate weighted fat suppressed sequences for semi-quantitative (SQ) MRI Osteoarthritis Knee Scores (MOAKS) scoring.

Quantitative cartilage thickness in the MAC (mm) was determined from manual cartilage segmentations performed by experienced readers with blinding to time point (Chondrometrics GmbH, Freilassing, Germany) [13]. MOAKS scoring was performed by an experienced radiologist (FWR; >10 years experience with MOAKS scoring) and included meniscal extrusion, meniscal tear, total number of bone marrow lesions (BMLs), and BML size; for these four parameters, the sum score of subregions affected by specific pathology in the MAC was used. MOAKS synovitis and effusion were scored as well. Further, in the patellofemoral (PF) joint, cartilage damage (size of cartilage loss as a% of the surface area, and% area that is full-thickness loss), BMLs (number and size), and osteophytes (size) were scored; the sum of all PF subregions was used [14].

### Statistical analysis

Two-year changes in gait parameters were compared between structural progressors and non-progressors. Progression cut-offs were the smallest detectable change (SDC) for all continuous measures or one full score for KL grade and MOAKS scores (published previously) [11,13,15]. Additionally, for minimum JSW, the structural progression definition as determined in the IMI-APPROACH study protocol before participant inclusion (two-year change of ≤−0.6 mm) was used [5,15].

To reduce data and the number of tests performed, principal component analysis (PCA) was performed on the two-year changes in gait parameters first. The resulting principal component was compared between progressors and non-progressors for all structural measures using independent *t*-tests. Since the principal component represents a linear combination of the four gait parameters, these values are not easily interpretable. As such, in case these results indicated differences between groups for a specific structural measure (*p* < 0.1), two-year changes in the four individual gait parameters were compared directly using independent *t*-tests. Significant results from these comparisons were additionally corrected for two-year WOMAC pain change in the index knee, using linear regression. Only participants with gait measurements and at least one type of image evaluation (radiographic, qMRI, or SQ MRI) at both baseline and two years of follow-up were included.

## Results

### Participants

In total, 191 participants were included; those not included most frequently did not have complete two-year data, mainly the result of COVID-19. Their demographics are provided in Supplementary Table S1. Most participants had a medial MAC (85 %), right index knee (59 %), and female sex (76 %). Half (49 %) did not have radiographic OA at baseline (KL grade 0 or 1).

Over two years, only knee ROM in swing phase showed a statistically significant decrease from 58.9 (SD 6.8) degrees at baseline to 57.1 (7.5) degrees at two years (*p* < 0.001); see Supplementary Table S2.

### Gait changes in progressors and non-progressors

PCA on the four gait parameters resulted in one principal component to be compared between structural progressors and non-progressors. For most pathologies, progressors showed gait deterioration while non-progressors did not (Table 1). Differences between progressors and non-progressors were in most cases not statistically significant except for MOAKS meniscal extrusion (*p* = 0.018) and MOAKS BML number (*p* = 0.045). Further, MOAKS% area cartilage loss in the PF compartment (PF_Area_) indicated that gait improved for progressors, while non-progressors deteriorated (*p* = 0.064).

**Table 1:** Two-year gait change (principal component) for structural progressors and non-progressors.

		Two-year change gait (principal component)		
Parameter	Progressors n (%)	Progressors mean (SD)	Non-progressors mean (SD)	P-value
*Predefined progression (minimum JSW change ≤−0.6mm over two years)*				
Minimum JSW	32 (17)	−0.10 (1.01)	0.01 (1.00)	0.581
*FT progression in the MAC (change ≥SDC or 1 full score)*				
Minimum JSW	43 (23)	−0.04 (1.00)	−0.00 (1.00)	0.818
FT Angle	47 (25)	0.19 (1.01)	−0.07 (1.00)	0.122
Subchondral bone density	79 (42)	−0.06 (0.98)	0.02 (1.01)	0.596
Osteophyte size	67 (35)	−0.14 (0.90)	0.06 (1.04)	0.194
qMRI cartilage thickness	68 (37)	−0.08 (1.07)	0.03 (0.97)	0.194
MOAKS meniscal extrusion	26 (14)	−0.43 (0.86)	0.06 (0.99)	**0.018**
MOAKS meniscal tear	23 (12)	−0.24 (1.00)	0.03 (1.00)	0.224
MOAKS BML number	21 (11)	−0.42 (1.12)	0.04 (0.97)	**0.045**
MOAKS BML size	22 (13)	−0.24 (1.06)	−0.00 (0.98)	0.301
*FT progression (change ≥1 full score)*				
MOAKS synovitis	15 (8)	0.06 (1.26)	−0.01 (0.98)	0.820
MOAKS effusion	45 (24)	0.19 (1.01)	−0.06 (1.00)	0.138
KL grade	26 (14)	−0.20 (1.18)	0.03 (0.97)	0.281
*PF progression (change ≥ 1 full score)*				
MOAKS% area cartilage loss	23 (14)	0.37 (1.17)	−0.05 (1.00)	*0.064*
MOAKS% full thickness loss	32 (19)	−0.03 (0.94)	0.01 (1.04)	0.850
MOAKS osteophyte size	13 (7)	−0.23 (0.71)	0.02 (1.01)	0.398
MOAKS BML number	30 (16)	0.12 (1.00)	−0.03 (1.00)	0.456
MOAKS BML size	19 (10)	0.03 (0.93)	0.03 (1.08)	0.991

Note that these values are for the principal component, linearly representing the four different gait parameters.

SD: standard deviation; JSW: joint space width; FT: femorotibial; MAC: most affected compartment; SDC: smallest detectable change; qMRI: quantitative MRI; MOAKS: MRI Osteoarthritis Knee Scores; BML: bone marrow lesion; KL: Kellgren-Lawrence.

For these three parameters, individual gait parameter changes between progressors and non-progressors are shown in Table 2. Participants with MOAKS meniscal extrusion progression showed significantly more worsening in knee ROM in the swing phase and calf ROM (both *p* < 0.04). Participants with MOAKS number of BMLs progression showed significantly more thigh ROM worsening (*p* = 0.045). For both pathologies, all other gait parameters show more worsening for progressors than for non-progressors as well, though not statistically significant (all *p* > 0.06). Controversially, participants with MOAKS PF_Area_ progression showed less gait worsening for all four gait parameters, which was significant for the knee ROM in stance phase (*p* = 0.039), and often even showed improvement.

**Table 2:** Changes in gait parameters for progressors and non-progressors in three selected pathologies.

	ROM Knee swing phase Mean change (SD), in degrees			ROM knee stance phase Mean change (SD), in degrees			ROM thigh Mean change (SD), in degrees			ROM calf Mean change (SD), in degrees		
Parameter	Progressors	Non-progressors	P-value	Progressors	Non-progressors	P-value	Progressors	Non-progressors	P-value	Progressors	Non-progressors	P-value
MOAKS meniscal extrusion	−4.9 (7.0)	−1.2 (6.9)	**0.012**	−0.6 (5.6)	−0.1 (5.4)	0.658	−1.5 (5.0)	0.4 (5.4)	0.094	−2.8 (5.5)	−0.4 (5.5)	**0.035**
MOAKS BML number	−3.6 (6.2)	−1.5 (7.0)	0.198	−2.3 (4.6)	0.0 (5.4)	0.064	−2.1 (6.0)	0.5 (5.4)	**0.045**	−1.8 (7.0)	−0.5 (5.5)	0.325
PF MOAKS% area cartilage loss	−0.5 (7.9)	−2.0 (6.7)	0.304	2.1 (5.3)	−0.5 (5.5)	**0.039**	0.7 (6.6)	0.2 (5.5)	0.713	1.5 (6.3)	−0.9 (5.7)	0.057

ROM: range of motion; SD: standard deviation; MOAKS: MRI Osteoarthritis Knee Scores; BML: bone marrow lesion; PF: patellofemoral.

All statistically significant differences between progressors and non-progressors in Table 2 were still significant even when adjusting for pain change, except for the difference in ROM thigh change between MOAKS BML number progressors and non-progressors (*p* = 0.051 instead of *p* = 0.045). However, the difference between groups barely changed after adjusting for pain change (*B*=−2.57 instead of *B*=−2.54).

## Discussion

This study revealed that worsening of both meniscal extrusion and number of BMLs were associated with gait worsening, whereas surprisingly worsening of MOAKS PF_Area_ (i.e. area extent of superficial cartilage damage) was associated with gait improvement. When accounting for change in pain, the association observed between BML progression and gait worsening after adjustment changed the p-value and group differences slightly.

We previously reported meniscal extrusion at baseline in IMI-APPROACH participants with radiographic OA to be associated with change in lower leg gait [3]. The current study extends the finding that not only the presence/severity of meniscal extrusion, but also further worsening is detrimental for gait alterations especially in the knee and calf, independent of pain. The main functions of the meniscus are femorotibial load transmission, shock absorption, joint stability and lubrication during gait [16]. A large study on knee OA patients concluded that meniscal extrusion is probably an effect of the complex interactions among joint tissues and mechanical stresses involved in the OA process [17]. It is therefore not unexpected that any changes to the menisci would have an effect on gait kinematics, although this was not the case for meniscal tears. The two-year change in knee ROM for the meniscal extrusion progressors was −4.9° (Table 2), against a total population baseline of 58.9° (Supplementary Table S2). Referring to the healthy population[8], the baseline is already 1σ below normal and the reduction results in >2σ away from healthy, which is a clinically significant difference. The knee stance ROM did not differ significantly, indicating that meniscal extrusion affects the knee movement in the swing phase, but less so in the loading phase.

In contrast to meniscal extrusion, BMLs have no direct biomechanical impact on gait, but have been shown to be associated with knee pain [18]. Thigh ROM is directly related to stride length, which patients reduce to minimize pain [19]. The association between the increase in the number of BMLs in the MAC and worsening of thigh ROM can therefore most likely be explained by the indirect impact of BMLs on gait via pain.

The gait improvement observed in participants with worsening of MOAKS PF_Area_ is surprising and may potentially be explained by these participants having more PF-driven instead of FT-driven OA, resulting in less FT progression and less gait deterioration. However, comparing PF_Area_ progression with progression in FT parameters shows similar distributions (chi-square tests all *p* > 0.2). Further, 13 (57 %) of PF_Area_ progressors did have FT radiographic OA (KL grade ≥2) at baseline. Cartilage lesion progression could, however, be the result of factors other than OA progression, such as patellar instability/dislocation or decreased PF loading, although this still does not explain why it would be associated with gait improvement [20,21]. These results should be evaluated in a larger cohort with more PF outcome measures, to confirm the association and further explore the mechanism.

Conservative OA treatments, such as pain medication, often address the symptoms that patients experience, but not the cause. However, these results indicate that to improve functional symptoms like gait, correcting the structural damage may be required as well. For gait specifically, meniscal extrusion and BMLs appear to be a relevant treatment target, to help patients increase their knee function. To date, degeneration and regenerative surgical treatments often focus on one structural characteristic, especially cartilage thickness. However, solely treating cartilage, even if it is combined with pain reduction, may not significantly impact gait, at least over the short term; meniscal extrusion and BMLs might be more important. A larger study to ascertain whether meniscal extrusion is indeed a more relevant pathology to evaluate against gait changes, for example after meniscal repair surgery, would be of interest. Surgical centralization procedures have been introduced to re-align the extruded meniscal body, but long term results on positive effect including possible impact on gait are missing to date [[22], [23], [24]]. Importantly, while we evaluated statistical relevance in this study, it was not considered whether gait changes were clinically relevant as well, since no minimal clinically important difference has been determined.

This study has several limitations. Only 191 participants could be included in the current study because of the limited overall sample size and, while PCA was performed to reduce the number of statistical tests, we did not correct for multiple testing. In addition, IMI-APPROACH was a two-year study and structural progression is a slow process, limiting the magnitude of evaluated changes and the ability to observe associations between change in structural parameters and gait. A considerable proportion of the IMI-APPROACH enrollees also had no definite radiographic OA. Still, a tendency towards worsening of gait was observed for the majority of structural parameters and future studies with longer observation periods and/or larger sample sizes may be able to provide additional insights into the impact of worsening of structural damage on gait. Lastly, WOMAC pain questionnaires were filled out in the days before the hospital visit where the gait analyses were performed, not on the same day. Hence, patients might have been in slightly more or less pain during their gait analysis than reflected in the WOMAC pain, although these differences are expectedly very small.

In conclusion, while the surprising positive effect of PF_Area_ progression on gait needs further investigation, meniscal extrusion and BML progression seem to be significantly associated with gait worsening in knee OA patients, although for BMLs this might be to be related to a pain increase. Further evaluation in clinical trials could show whether preventing progression or treating these pathologies could directly lead to functional gait improvement in patient suffering from knee OA.

## Declaration of competing interest

DH: founder and CEO of Dynamic Metrics Ltd, owner of GaitSmart.

MK: consulting fees from Abbvie, Pfizer, Kiniksa, Flexion, Galapagos, Jansen, CHDR, Novartis, UCB, all paid to institution.

FJB: funding from Gedeon Richter Plc., Bristol-Myers Squibb International Corporation (BMSIC), Sun Pharma Global FZE, Celgene Corporation, Janssen Cilag International N.V, Janssen Research & Development, Viela Bio, Inc., Astrazeneca AB, UCB BIOSCIENCES GMBH, UCB BIOPHARMA SPRL, AbbVie Deutschland GmbH & Co.KG, Merck KGaA, Amgen, Inc., Novartis Farmacéutica, S.A., Boehringer Ingelheim España, S.A, CSL Behring, LLC, Glaxosmithkline Research & Development Limited, Pfizer Inc, Lilly S.A., Corbus Pharmaceuticals Inc., Biohope Scientific Solutions for Human Health S.L., Centrexion Therapeutics Corp., Sanofi, TEDEC-MEIJI FARMA S.A., KiniksaPharmaceuticals, Ltd. Grunenthal

IKH: Research grant (ADVANCE) from Pfizer (payment to institution) and consulting fees from Novartis, Grünenthal and GSK, outside of the submitted work.

FB: Institutional grants from TRB Chemedica and Pfizer. Consulting fees from AstraZeneca, Boehringer Ingelheim, Heel, Galapagos, Gilead, Grunenthal, GSK, Eli Lilly, MerckSerono, MSD, Nordic Bioscience, Novartis, Pfizer, Roche, Sandoz, Sanofi, Servier, UCB, 4P Pharma. Honoraria for lectures from Pfizer, Eli Lilly, Viatris. Payment for expert testimony from Pfizer and Eli Lilly. Travel support from Nordic Pharma, Pfizer, Eli Lilly, Novartis. Stock owner and CMO of 4Moving Biotech.

FE: CEO and shareholder of Chondrometrics GmbH, received personal fees from TrialSpark, 4P Pharma, Norvartis, Servier, Galapagos and Merck and grants from PMU, BMBF, EU, NIH, FNIH, Merck, Galapagos NV, Novartis, TissueGene, CALIBR, ICM, Bioclinica/Clario, Universities of Erlangen Sydney, Basel Western Ontario, Stanford and Utrecht.

FWR: shareholder of Boston Imaging Core Lab (BICL), LLC and consultant to Calibr and Grünenthal.

WW: Employee and shareholder of Chondrometrics GmbH and consulting fees from Galapagos NV. The other authors have nothing to disclose.

## Appendix Supplementary materials

Image, application 1
