# Supplementary material for: Association between progression of knee osteoarthritis pathology and gait changes over two years: Data from the IMI-APPROACH cohort
Source: Osteoarthr Imaging. 2024 Jun 10;4(3):100232. doi: 10.1016/j.ostima.2024.100232 (PMC13228702; doi:10.1016/j.ostima.2024.100232)
Supplement: Supplementary file 1 [file mmc1.docx]

Supplementary Table S1: Baseline demographics of the 191 included participants.

| **Parameter** | **Mean ± SD or n (%)** |
| --- | --- |
| Age | 66.5 (6.7) |
| Male sex | 45 (24) |
| BMI | 27.5 (4.8) |
| KL grade |  |
| - 0 | 34 (18) |
| - 1 | 60 (31) |
| - 2 | 38 (20) |
| - 3 | 51 (27) |
| - 4 | 8 (4) |
| Right index knee | 112 (59) |
| Medial MAC | 162 (85) |

BMI: body mass index; KL: Kellgren-Lawrence; MAC: most affected compartment.

Supplementary Table S2: Changes in gait parameters and pain over two years.

| **Parameter** | **Baseline** | **Two years** | **Change** | **P-value** |
| --- | --- | --- | --- | --- |
| ROM knee swing phase | 58.9 (6.8) | 57.1 (7.5) | -1.7 (6.9) | **<0.001** |
| ROM knee stance phase | 16.2 (5.0) | 15.9 (5.7) | -0.2 (5.4) | 0.576 |
| ROM thigh | 37.2 (5.8) | 37.4 (5.4) | 0.2 (5.5) | 0.598 |
| ROM calf | 72.5 (6.2) | 71.9 (6.9) | -0.6 (5.7) | 0.118 |
| WOMAC pain | 70.2 (19.6) | 70.5 (20.8) | 0.4 (15.2) | 0.740 |

ROM: Range of motion; WOMAC: Western Ontario and McMaster Universities Osteoarthritis Index.
